# Empowering smokers with a web-assisted tobacco intervention to use prescription smoking cessation medications: a feasibility trial

**DOI:** 10.1186/s13012-015-0329-7

**Published:** 2015-10-01

**Authors:** Peter Selby, Sarwar Hussain, Sabrina Voci, Laurie Zawertailo

**Affiliations:** Addictions Program, Centre for Addiction and Mental Health, 100 Stokes St., Toronto, ON M6J 1H4 Canada; Department of Family and Community Medicine, University of Toronto, 500 University Avenue, Toronto, ON M5G 1V7 Canada; Department of Psychiatry, University of Toronto, 250 College Street, 8th floor, Toronto, ON M5T 1R8 Canada; Dalla Lana School of Public Health, University of Toronto, 155 College Street, Toronto, ON M5T 3M7 Canada; Ontario Tobacco Research Unit, 155 College Street, Toronto, ON M5T 3M7 Canada; Department of Pharmacology and Toxicology, University of Toronto, 1 King’s College Circle, Toronto, ON M5S 1A8 Canada

**Keywords:** Tobacco, Smoking, Smoking cessation, Bupropion, Varenicline, Open-label, Web-assisted tobacco intervention, Pilot study, Feasibility study

## Abstract

**Background:**

Varenicline and bupropion, efficacious smoking cessation medications, have had suboptimal impact due to barriers at the patient, practitioner and system level. This study explored the feasibility of a web-assisted tobacco intervention offering free prescription smoking cessation medication by mail if the smoker visited a physician for authorization.

**Methods:**

Adult Ontarians, smoking at least 10 cigarettes daily, intending to quit within 30 days, with no contraindications to bupropion or varenicline were eligible. After an online assessment, eligible participants received an electronic personalized printable prescription form for a 12-week course of varenicline or bupropion to bring to a physician within 3 weeks for authorization, if appropriate. The physician’s office faxed prescriptions to an online pharmacy that couriered medication to the patient following medication counselling by telephone. Weekly motivational emails were sent during treatment. Participants were asked to complete follow-up questionnaires online at 7, 11, 15 and 41 weeks after enrollment.

**Results:**

In total, 1214 individuals submitted an online assessment from April to September 2010 and 73.6 % (95 % confidence interval (CI) = 71.1–76.1 %; *n* = 893) were eligible. At least 65.8 % (95 % CI = 62.7–68.9 %; *n* = 588) of eligible participants subsequently visited a physician and 58.7 % (95 % CI = 55.5–61.9 %; *n* = 524) received medication (50.6 % varenicline [*n* = 265] and 49.4 % bupropion [*n* = 259]). Reasons for not filling a prescription were failure to visit a physician (80.1 %; 95 % CI = 73.8–86.5 %; *n* = 121), physician not prescribing the medication (15.9 %; 95 % CI = 10.1–21.7 %; *n* = 24) or other reasons (4.0 %; 95 % CI = 0.9–7.1 %; *n* = 6). Follow-up response rate was 66.7 % (95 % CI = 63.7–69.8 %; *n* = 596). Minimal issues were encountered with printing the prescription or medication delivery.

**Conclusions:**

This study establishes the feasibility of using the Internet and free medication to enable smokers to engage physicians to treat this addiction. Implementation of this intervention can be scaled up by leveraging existing healthcare systems to treat smokers on a population level. Further evaluation in a randomized controlled trial is necessary.

**Trial registration:**

ClinicalTrials.gov Identifier NCT01023659

**Electronic supplementary material:**

The online version of this article (doi:10.1186/s13012-015-0329-7) contains supplementary material, which is available to authorized users.

## Background

Each year, approximately six million people worldwide die prematurely due to tobacco-related diseases [1]. For each person who dies from a smoking-related disease, approximately 30 more people have at least one serious medical condition caused by smoking [2]. The majority of smokers want to quit [3, 4] but find it difficult [5]. Each year, close to half of smokers in North America make a quit attempt [4, 6], but without assistance only 3–5 % achieve long-term abstinence [7]. Several smoking cessation medications have been shown to improve long-term abstinence rates [8, 9], but despite their proven efficacy, approximately two-thirds of smokers report not having used medication in their most recent quit attempt [10].

Barriers to the use of these medications exist at the patient, system and practitioner level. Patient-level barriers include poor awareness, perceived lack of effectiveness, low desirability including concern regarding adverse effects, and poor accessibility of treatment [11, 12]. Accessibility issues include anticipated difficulties obtaining a prescription from a physician and perceived high cost of medication [11]. Full coverage of the cost of smoking cessation medications has been proven to increase utilization and improve quit rates [13, 14]. Advice by a healthcare provider to quit smoking has also been associated with increased use of smoking cessation treatments [15]. Though evidence-based clinical practice guidelines recommend that every smoker be advised to quit and assisted if ready to make a quit attempt [16, 17], a recent Canadian survey found that of the smokers who visited a physician in the previous 12 months, only 56 % received advice to quit and 34 % received information about smoking cessation aids [4].

Passive dissemination of guideline recommendations is generally ineffective in changing practitioner behaviour, and more intensive (and costly) training is generally necessary to alter practice [18]. Strategies to promote implementation of smoking cessation guidelines are most often targeted to clinicians [19]. Healthcare professionals who have received training for delivery of smoking cessation interventions are more likely to perform cessation-related tasks such as asking patients to set a quit date, offer counselling and provide self-help materials [20]. However, a Cochrane review found no demonstrated effect of training on provision of smoking cessation medications [20]. Therefore, new methods are required to enhance the prescribing of these evidence-based medications, which almost double to triple the odds of long-term cessation [8]. There is evidence that interventions targeted to patients can also change clinician practice and improve care [19, 21]. Patient-mediated interventions are delivered to patients with the aim of changing the attitudes, knowledge, skills and/or behaviour of the healthcare provider via patient-provider interaction [22]. Empowering patients to take an active role in their treatment in this manner may help foster greater autonomous motivation to use smoking cessation medications, which is associated with increased medication use, and in turn, higher levels of abstinence [23].

Large-scale distribution of free over-the-counter nicotine replacement therapies (NRT) mailed to smokers wanting to quit has had demonstrated success and reduces the barrier of geographic access to health services [24, 25]. However, regulatory restrictions require a visit to a prescriber to obtain a prescription for bupropion or varenicline. The current study was conducted to test the feasibility of a web-assisted tobacco intervention designed to increase the use of prescription smoking cessation medications by having patients prompt a physician to prescribe medication. Smokers motivated to quit were directly engaged online and were required to visit their physician within 3 weeks with a pre-populated form that was converted to a prescription upon physician signature in order to receive a free 12-week supply of bupropion or varenicline, mailed to them by an online pharmacy. The offer of free medication motivated the patient to initiate the dialogue with their physician. The online pharmacy was able to courier medication to any jurisdiction to increase the reach of the intervention and make it more accessible to those living in rural and remote areas. Given that this design is innovative and involves multiple steps with different providers, it was important to establish feasibility prior to conducting a larger-scale randomized clinical trial [26, 27]. We hypothesized that this intervention would be a logistically feasible approach to provide prescription medication to a large number of smokers over a wide geographic area.

## Methods

### Study design

An open-label trial was conducted to test the feasibility of distributing by mail a free 12-week supply of bupropion SR (Zyban) or varenicline (Champix, Chantix) to smokers following a visit to a physician of their choice for authorization of the prescription, in conjunction with weekly motivational emails and with a 41-week follow-up period to assess quit outcomes. The study was approved by the Research Ethics Board at the Centre for Addiction and Mental Health and was registered as a clinical trial (ClinicalTrials.gov Identifier NCT01023659).

### Participant recruitment

Adult smokers from Ontario, Canada, interested in quitting could enroll at the study website (www.stopstudy.ca; see Additional file 1). Study information, including an electronic copy of a recruitment poster that could be printed and posted, was provided to healthcare professionals from public health units, community health centres, family health teams and community pharmacies across Ontario. Physicians were also informed about the study via the Ontario College of Family Physicians newsletter.

Inclusion criteria required that participants be 18 years of age or older, residents of Ontario, smoke at least 10 cigarettes per day, had smoked daily for at least the past 3 months, had smoked at least 100 cigarettes in their lifetime and intended to quit smoking within the next 30 days. Exclusion criteria were self-reported: history of brain injury or seizure(s); pregnancy, lactation or risk of becoming pregnant; allergy to bupropion or varenicline; current use of monoamine oxidase inhibitors (MAOIs), thioridazine, varenicline or bupropion; severe liver impairment; or history of anorexia and/or bulimia. The inclusion criteria were to mimic efficacy trial inclusion criteria. The exclusion criteria were those listed as absolute exclusions in the product monograph for bupropion and varenicline at the time of the study.

### Sample size

The number of participants enrolled in each group was determined by the quantity of each medication available for this study, considered sufficient for achieving the primary goal of determining the logistic feasibility of the proposed distribution method. With a sample size of approximately 500, we could estimate a physician visit rate of 50 % to within a 95 % confidence interval of ±4 %.

### Intervention

**Fig. 1:**
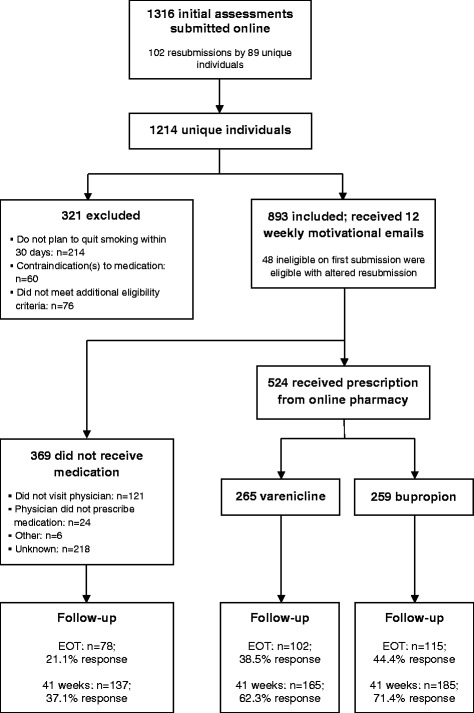
Flow chart of recruitment and follow-up. *EOT* end of treatment (15 weeks after study enrollment)

A diagram outlining the participant flow is provided in Fig. 1.

#### Safety considerations

A number of checks were incorporated into the design to ensure that distribution was executed safely. The first check was excluding participants who reported any one of several contraindications to either medication during the initial online assessment; this also minimized resources from being wasted due to unnecessary physician appointments. Should an individual have met eligibility criteria by either deliberately or inadvertently submitting an incorrect response, the physician acted as a secondary check to screen for contraindications prior to prescribing the medication. Finally, the pharmacist acted as third safety check by screening for potential drug interactions and fraudulent prescriptions.

#### Online self-assessment and automated eligibility determination

Prior to being asked to provide any personal information, participants were required to provide informed consent by selecting the “Yes” option to indicate their agreement with the statement of consent. The website presented frequently asked questions and contact information of study personnel if individuals had additional questions about study participation. The initial assessment questions were designed to assess eligibility and collect information on demographic and baseline smoking characteristics. Participants were required to provide an email address at which they could be contacted regarding their eligibility and for follow-up. Individuals were not able to submit more than one assessment using the same email address. Upon submitting the initial assessment, an automated process informed participants via email whether or not they were eligible. Those not eligible were provided with a list of smoking cessation resources they could access for assistance with quitting.

#### Personalized prescription form

Eligible smokers were immediately sent two documents via email (see Additional file 2) with instructions to print and bring to their physician within 3 weeks of study enrollment, after which the prescription would be void. We estimated that 3 weeks would allow time to get an appointment with a physician and still receive medication within the 30-day period during which participants were planning to quit. The documents were as follows: (1) a letter to the physician that provided information about the study and (2) a personalized prescription form for study medication. The patient name and address fields were automatically filled in on the prescription form based on information entered in the initial assessment. The prescription form provided the option to select either bupropion (150 mg once daily for 3 days, then 150 mg twice daily for the remainder of 12 weeks) or varenicline (0.5 mg once daily for 3 days, 0.5 mg twice daily for 4 days, then 1.0 mg twice daily for the remainder of 12 weeks). Information about the potential side effects and risks of each medication were presented to the participant prior to them providing informed consent. Given that the quantity of each medication was limited, when the supply of one type of medication was depleted, the option of selecting either medication was removed from the website and on the prescription form. The email to eligible participants also contained personal login information for the study website where they could access both documents at any time.

#### Patient visit to physician

In order to receive medication, participants were required to schedule a visit with a physician of their choice to whom they would provide both study documents for review, clarification and final determination of eligibility. The letter explicitly stated that the physician had full discretion to prescribe either medication to the patient and that “the study intends to fully defer to the patient-doctor relationship and thus leave the patient under your clinical care” (see Additional file 2). There were no incentives provided to the physician but they were free to bill the public healthcare system (Ontario Health Insurance Plan) for the visit as per their respective eligibility and compensation model. To prescribe either medication, the physician could checkmark the appropriate box on the prescription form to indicate the type of medication (if a choice was still available) and authorize the form by providing the date, their name, signature and registration number. If either medication was prescribed, the physician’s office was required to fax the signed prescription form to the online pharmacy (www.pharmacy.ca). The form included explicit instructions and the pharmacy name, fax and phone number.

#### Online pharmacy

The pharmacist verified the authenticity of the prescription and contacted the physician’s office if any of the information on the form was incomplete or missing as per their usual prescription verification practices. In accordance with the Ontario College of Pharmacists’ standard of practice, each participant was contacted by a pharmacist when dispensing medication to discuss possible allergies, concomitant medications and provide relevant medication counselling. Participants were cautioned regarding the possibility of drowsiness and to avoid driving or operating machinery until they were reasonably certain that the medication did not affect their mental alertness or physical coordination. The pharmacist asked the participant to confirm their address and then couriered the medication to the participant directly. An online pharmacy allowed participants to receive their medication without the additional step of visiting a pharmacy in person for dispensing, thereby eliminating a potential barrier.

#### Behavioural support

All participants deemed eligible after completing the initial assessment, regardless of whether they received medication or not, were sent a weekly motivational email for 12 weeks. Emails commenced at 3 weeks after study enrollment, the date by which prescriptions had to be submitted for medication dispensing. See Table 1 for examples of email messages sent. Each medication was accompanied by a self-help booklet provided by the manufacturer that included smoking cessation tips and how to access web-based or telephone counselling support.

**Table 1:** Examples of weekly motivational email messages

Weekly tip number	Tip
1	Creating a smoke-free environment is important during your quit attempt. Make a decision not to smoke in your home and vehicle and ask others to do the same. If your entire home cannot go smoke-free, explore areas where you can restrict smoking. At work, avoid smoking areas during your breaks. Making your physical environment smoke-free can help reinforce your decision to quit smoking.
2	Support systems are important during any big change. Identify all of the positive supports in your life and tell them you are quitting smoking and need their support. Also identify any negative influences who may not want you to quit and figure out how you are going to deal with them during this time. Take advantage of other supports available to you, such as Smoker's Helpline, websites, your doctors or other healthcare providers. Surrounding yourself with positive and supportive people can help you quit and stay quit.
3	Slips and lapses are a part of the quitting process and can be common. Use any slip or lapse as a learning experience. Identify what happened, how you could have prevented the situation, and what you can do if you’re in the situation again. Use these experiences to re-assess your quit plan and then try quitting again. It is important that you realize your quit attempt is not over; refocus and restart immediately after your lapse. Remember, quitting smoking is a process not an event and may take several attempts before you get it right.
4	One of the benefits of quitting smoking is the amount of money you save. The price of a pack of cigarettes is about C$8; so, that means if you smoked about 15 cigarettes every day you would save about C$570 in 3 months (enough to purchase a new 32-inch flat-screen TV) or save C$2180 in 1 year (enough for a long vacation abroad). Therefore, take advantage of quitting smoking and reward yourself.
6	Your smoking may be associated with certain people, places, or things. These can act as triggers for you to want to smoke. Identify your personal triggers and think about how you will deal with them. For example, change your day-to-day routine or find alternative activities to smoking. Problem-solving ahead of time can help you deal with these situations when they arise and help you quit and stay quit.
8	There are many good reasons why people want to quit smoking. Sometimes it’s easy to forget why you wanted to quit in the first place. Write down your personal reasons for quitting and use them as reminders when things seem tough. Your reasons may change over time so review your list regularly. Reminding yourself of all the reasons you want to quit can help you stay focused on achieving your goal.

### Participant follow-up

Collection of follow-up data was attempted via email for all participants at 7, 11, 15 and 41 weeks after enrollment. We estimated that medications would be started approximately 3 weeks after enrollment. Monthly contact for the 12-week period thereafter was to monitor any side effects and assess quit success. Follow-up at 41 weeks after enrollment (approximately 6 months after the end of treatment) was to assess long-term quit success. At each follow-up, participants were screened for suicidal ideation because of post-marketing concerns regarding both bupropion and varenicline. Participants who screened positive were advised to stop their medication and contact their physician immediately or report to their nearest Emergency Department. One reminder email was sent to non-respondents. Participants were also provided a telephone number with the option of completing the survey via telephone.

### Outcome measures

The primary feasibility outcome was that at least 50 % of eligible smokers planning to quit within 30 days would report being able to schedule an appointment with a physician before the prescription expired at 3 weeks after enrollment. A 2008 survey found that 68 % of all adults in Canada report being able to get an appointment with a physician within 7 days and 89 % within 30 days for routine care [28]. Based on this finding, we estimated that at least 75 % of participants would be able to schedule an appointment with a physician within 3 weeks after enrollment. Taking into consideration that potential delays in calling the physician’s office (e.g. varying levels of motivation and failure to remember) and conflict with other responsibilities and priorities (e.g. employment and childcare) may lower this number, we set 50 % as the threshold for feasibility. A low proportion of physician visits was considered acceptable in light of the negligible costs associated with enrolling and following up with participants who do not visit a physician. The following cut-off points were also set in order to establish the logistic feasibility of the distribution method prior to proceeding with a larger study: (1) ability to recruit a sample of approximately 500 participants within 6 months without using paid advertisements, based approximately on the recruitment rate from a study we conducted distributing free NRT that required at least one visit to a pharmacy [29], assuming one quarter as many individuals would be interested in prescription cessation medications compared to NRT [30] and a lower eligibility rate due to a greater number of contraindications associated with bupropion and varenicline; (2) eligibility rate of at least 70 %, similar to comparable randomized trials comparing bupropion and varenicline [31, 32]; (3) at least 45 % of eligible participants would receive medication, based on the assumption that at least 50 % are able to visit a physician, and of these, almost all would receive a prescription due to the exclusion of those with contraindications at the initial assessment; and (4) no more than 7 days would be typically required to reach the participant via telephone for medication counselling and to confirm shipping address, operationalized as the median number of days between filling the prescription and sending the medication out for delivery, permitting most participants to receive medication within 30 days.

Additional feasibility data collected included reasons for not scheduling a visit with a physician, frequency of medication delivery issues, and the average number of days between study enrollment and prescription dispensing and delivery. Logistic feasibility outcome data were collected from participants at each follow-up survey (to maximize the probability of having feasibility data for each participant, given loss to follow-up) and also extracted from pharmacy records. Where a participant provided conflicting responses at different follow-up time points with respect to feasibility outcomes, the earliest response is reported based on the assumption that recall would be less accurate as time elapsed.

Acceptability of the motivational email component of the intervention was assessed by asking participants at each follow-up throughout the 12-week treatment period to rate the helpfulness of the emails on a five-point scale from 1 (not helpful at all) to 5 (very helpful). Responses provided later in treatment replaced ratings provided earlier in treatment, where applicable. Participants were also asked throughout and at the end-of-treatment follow-up how much of the 12-week supply of medication they had used. This was done because completion of treatment is a predictor of successful quitting [33]. Participants were asked to select from several response options provided, which were collapsed into a smaller number of categories for analysis (none, less than 1 week, 1 to less than 4 weeks, 4 to less than 8 weeks, 8 to less than 12 weeks, all 12 weeks). As above, responses provided further along in treatment replaced earlier responses, where applicable.

Treatment outcome was assessed in order to obtain an estimate of treatment effect to inform a sample size calculation for a future randomized trial. The outcome variable related to the effectiveness of the intervention was 7-day point prevalence of abstinence from smoking at 41 weeks after enrollment, approximately 6 months after the individual completed their 12-week course of medication. Seven-day point prevalence abstinence was defined as not having smoked even a puff over the previous 7 days.

### Statistical analysis

Comparisons on baseline variables were made using chi-square tests of association for categorical variables and *t* tests for continuous variables to detect any differences between participants who received medication and those who did not, as well as between those who received bupropion versus varenicline. Respondents and participants lost to follow-up at 41 weeks were similarly compared on baseline characteristics to identify potential attrition bias, and a comparison on baseline demographic characteristics was made for participants who were able to visit a physician versus those who were not. A one-sample chi-square test was used to compare the observed versus expected frequencies of smokers in each geographic region based on population estimates derived from the 2009–2010 Canadian Community Health Survey [34].

Data screening prior to analysis revealed that the proportion of missing data values at baseline was highest for annual household income (8 %) and employment status (3 %); all other baseline variables had less than 1 % missing data. Cases with missing data were deleted pairwise in all bivariate analyses. To attempt to minimize attrition bias, multiple imputation was used to address the missing data for the analysis of the primary smoking cessation outcome at 41-week follow-up; findings are presented alongside a complete case analysis. Multiple imputation by chained equations was implemented using Stata 12 (mi impute chained) to generate 20 imputed datasets, with a burn-in period of 10 iterations. An inclusive strategy was used to impute data in an attempt to maximize efficiency and reduce bias [35]; thus, additional auxiliary variables from the baseline questionnaire were included in the imputation model (e.g. number of cigarettes per day, the longest previous quit attempt, education level and employment status). Multiple imputation analyses were conducted with Stata 12 [36]; all other analyses were conducted with SPSS 15.0 [37]. Level of significance was set at *p* < 0.05, with Bonferroni adjustment to correct for multiple comparisons on baseline variables.

## Results

### Participants and recruitment

A total of 1214 unique individuals submitted an initial assessment between April and September 2010 (see Fig. 1). To manage and ensure availability of the limited supply of medication, enrollment was closed periodically for varying lengths of time to monitor how many eligible participants were filling prescriptions for each medication. Enrollment was highest in the first few months, with 80 % recruitment by the end of June. The median number of eligible participants that enrolled in the study on days that enrollment was open was 12 (interquartile range (IQR) 6–26) until the end of June and dropped to 4 (IQR 3–6) thereafter. Participants reported hearing about the study from the following sources: friends or family, 39.2 % (95 % confidence interval (CI) = 36.0–42.4 %; *n* = 348); physician, 16.8 % (95 % CI = 14.3–19.3 %; *n* = 149); public health unit, 15.1 % (95 % CI = 12.8–17.5 %; *n* = 134); other healthcare professional/organization, 5.6 % (95 % CI = 4.1–7.2 %; *n* = 50); media (newspaper, television and radio), 12.6 % (95 % CI = 10.4–14.8 %; *n* = 112); the Internet, 4.1 % (95 % CI = 2.8–5.4 %; *n* = 36); poster or flyer, 2.6 % (95 % CI = 1.5–3.6 %; *n* = 23); others, 3.2 % (95 % CI = 2.0–4.3 %; *n* = 28); and don’t know/refuse, 0.8 % (95 % CI = 0.2–1.4 %; *n* = 7).

Of the 1214 that submitted an initial assessment, 893 participants were eligible (73.6 %; 95 % CI = 71.1–76.1 %) and successfully enrolled in the study. Individuals were excluded for the following reasons: not planning to quit within 30 days, 17.6 % (95 % CI = 15.5–19.8 %; *n* = 214); smoked fewer than 10 cigarettes/day, 4.5 % (95 % CI = 3.4–5.7 %; *n* = 55); did not smoke daily over the past 3 months, 2.1 % (95 % CI = 1.3–2.9 %; *n* = 25); did not smoke cigarettes, 0.6 % (95 % CI = 0.2–1.0 %; *n* = 7); had smoked fewer than 100 cigarettes in their lifetime, 0.1 % (95 % CI = 0.0–0.2 %; *n* = 1); pregnant, 0.4 % (95 % CI = 0.1–0.8 %; *n* = 5); history of brain injury, 0.8 % (95 % CI = 0.3–1.3 %; *n* = 10), seizure, 1.4 % (95 % CI = 0.7–2.1 %; *n* = 17), bulimia or anorexia, 0.2 % (95 % CI = 0.0–0.4 %; *n* = 2) or allergic reaction to bupropion or varenicline, 0.7 % (95 % CI = 0.2–1.1 %; *n* = 8); or current use of bupropion, varenicline, MAOIs or thioridazine, 1.5 % (95 % CI = 0.8–2.2 %; *n* = 18).

Participant baseline characteristics are presented in Table 2. Participants who received either medication were older, had been smoking daily for longer and were less likely to report that all or most of their friends smoked. There were no statistically significant differences at baseline between participants who received bupropion versus varenicline. The proportion of the sample from each geographic region differed significantly from the proportions expected based on population estimates of smoking prevalence in each region [34], *χ*^2^ (*df* = 4) = 167.02, *p* < 0.001. In particular, the proportion of smokers from the city of Toronto was lower than expected (6.0 % [95 % CI = 4.4–7.5 %; *n* = 53] vs. 17.5 %) and the proportion of smokers from Southwestern Ontario was higher (25.1 % [95 % CI = 22.2–27.9 %; *n* = 223] vs. 13.7 %); differences were minimal (<5 %) in the remaining regions.

**Table 2:** Participant characteristics at baseline

Characteristic	Received medication		*p* ^a^	Type of medication		*p* ^a^
	No (*n* = 365)	Yes (*n* = 522)		Bupropion (*n* = 258)	Varenicline (*n* = 264)	
Female, *n* (%)	196 (53.7)	307 (58.8)	0.130	150 (58.1)	157 (59.5)	0.758
Age (years), mean (SD)	38.6 (12.0)	44.2 (12.5)	<0.001	44.4 (12.2)	44.0 (12.8)	0.729
Education level below high school diploma, *n* (%)	94 (25.9)	117 (22.4)	0.232	59 (22.9)	58 (22.0)	0.806
Annual household income ≤$20,000, *n* (%)	164 (48.8)	209 (42.9)	0.095	116 (46.4)	93 (39.2)	0.111
Employed, *n* (%)^b^	198 (54.2)	274 (52.5)	0.642	136 (52.7)	138 (52.3)	0.647
Geographic region, *n* (%)			0.495			0.418
Northern Ontario	46 (12.6)	50 (9.5)		26 (10.0)	24 (9.1)	
Toronto	23 (6.3)	30 (5.7)		19 (7.3)	11 (4.2)	
Central Ontario (excluding Toronto)	147 (40.3)	237 (45.2)		109 (42.1)	128 (48.3)	
Eastern Ontario	57 (15.6)	76 (14.5)		37 (14.3)	39 (14.7)	
Southwestern Ontario	92 (25.2)	131 (25.0)		68 (26.3)	63 (23.8)	
Cigarettes per day, *n* (%)			0.365			0.764
10–19	35.9 (131)	32.2 (168)		31.4 (81)	33.0 (87)	
20–29	51.0 (186)	51.9 (271)		51.6 (133)	52.3 (138)	
30+	13.2 (48)	15.9 (83)		17.1 (44)	14.8 (39)	
Time to first cigarette in the morning ≤5 min, *n* (%)	51.0 (186)	46.0 (240)	0.291	47.3 (122)	44.7 (118)	0.817
Years since started smoking daily, mean (SD)	24.2 (12.0)	29.9 (12.4)	<0.001	29.7 (12.1)	30.0 (12.7)	0.835
Fewer than three past quit attempts, *n* (%)^c^	220 (60.3)	270 (51.7)	0.012	140 (54.3)	118 (45.7)	0.251
Confidence in quitting (1–10), mean (SD)	7.5 (2.1)	7.8 (2.1)	0.019	7.8 (2.2)	7.8 (2.0)	0.865
Previous use of smoking cessation medication, *n* (%)^d^	186 (51.0)	284 (54.4)	0.311	142 (55.0)	142 (53.8)	0.774
Longest period of previous abstinence from smoking <1 week, *n* (%)	115 (31.5)	149 (28.5)	0.342	75 (29.1)	74 (28.0)	0.793
All or most friends smoke, *n* (%)	180 (49.3)	164 (31.4)	<0.001	80 (31.0)	84 (31.8)	0.749
Lives with other smoker(s), *n* (%)	219 (60.0)	279 (53.4)	0.053	140 (54.3)	139 (52.7)	0.712
History of drug or alcohol problem, *n* (%)	55 (15.1)	67 (12.8)	0.342	40 (15.5)	27 (10.2)	0.072

*N* varies due to missing data. All baseline data are missing for six participants

^a^The Bonferroni-adjusted threshold for significance was *p* < 0.003

^b^Employed full-time, part-time or self-employed

^c^Abstained from smoking for at least 24 h in an attempt to quit smoking

^d^Nicotine patch, gum, inhaler, varenicline, bupropion

### Follow-up response rates

The response rates at follow-up were as follows: 19.6 % (95 % CI = 17.0–22.2 %; *n* = 175) at 7 weeks; 18.7 % (95 % CI = 16.1–21.3 %; *n* = 167) at 11 weeks; 33.0 % (95 % CI = 29.9–36.1 %; *n* = 295) at 15 weeks (end of treatment); and 54.5 % (95 % CI = 51.3–57.8 %; *n* = 487) at 41 weeks. The response rate at 41-week follow-up among those that did not receive medication was substantially lower (37.1 %; 95 % CI = 32.2–42.1 %; *n* = 137) compared to the bupropion (71.4 %; 95 % CI = 65.9–76.9 %; *n* = 185) and varenicline (62.3 %; 95 % CI = 56.4–68.1 %; *n* = 165) groups. Overall, 66.7 % (95 % CI = 63.7–69.8 %; *n* = 596) of the sample had follow-up data for at least one time point. Several differences emerged between respondents and those lost to follow-up at 41 weeks (see Table 3). Respondents were older and had a higher annual household income; while they had been smoking daily for a greater number of years, they also reported longer periods of previous abstinence from smoking, reported a longer time to first cigarette in the morning and had a smaller proportion of friends who smoked.

**Table 3:** Comparison of baseline characteristics for respondents and participants lost to follow-up at 41 weeks

Characteristics	Enrolled (*N* = 887)^a^		
	Respondents (*n* = 505)	Lost to follow-up at 41 weeks (*n* = 382)	*p* ^b^
Female, *n* (%)	287 (59.3)	216 (53.6)	0.088
Age (years), mean (SD)	43.95 (12.47)	39.46 (12.35)	<0.001
Education level below high school diploma, *n* (%)	98 (20.3)	113 (28.1)	0.007
Annual household income ≤$20,000, *n* (%)	178 (39.7)	195 (52.0)	<0.001
Employed, *n* (%)^c^	256 (54.2)	216 (55.1)	0.799
Geographic region, *n* (%)			0.980
Northern Ontario	62 (12.8)	54 (13.4)	
Greater Toronto Area (GTA)	52 (10.7)	41 (10.2)	
Central Ontario (excluding GTA)	119 (24.6)	104 (25.8)	
Eastern Ontario	96 (19.8)	75 (18.6)	
Southwestern Ontario	155 (32.0)	129 (32.0)	
Cigarettes per day, *n* (%)			0.218
10–19	174 (36.0)	125 (31.0)	
20–29	245 (50.6)	212 (52.6)	
30+	65 (13.4)	66 (16.4)	
Time to first cigarette in the morning ≤5 min, *n* (%)	209 (43.2)	217 (53.8)	0.002
Years since started smoking daily, mean (SD)	27.19 (12.27)	22.80 (12.34)	<0.001
Fewer than three past quit attempts, *n* (%)^d^	253 (52.3)	237 (58.8)	0.051
Confidence in quitting (1–10), mean (SD)	7.74 (2.13)	7.60 (2.07)	0.303
Previous use of smoking cessation medication, *n* (%)^e^	262 (54.1)	208 (51.6)	0.454
Longest period of previous abstinence from smoking <1 week, *n* (%)	120 (24.8)	144 (35.7)	<0.001
All or most friends smoke, *n* (%)	157 (32.4)	187 (46.4)	<0.001
Lives with other smoker(s), *n* (%)	258 (53.3)	240 (59.6)	0.062
History of drug or alcohol problem, *n* (%)	58 (12.0)	64 (15.9)	0.093

^a^ *N* varies due to missing data. All baseline data were missing for six participants

^b^The Bonferroni-adjusted threshold for significance was *p* < 0.003

^c^Employed full-time, part-time or self-employed

^d^Abstained from smoking for at least 24 h in an attempt to quit smoking

^e^Nicotine patch, gum, inhaler, varenicline, bupropion

### Feasibility and acceptability outcomes

Among the entire sample with follow-up data, 79.7 % (95 % CI = 76.1–83.3 %; *n* = 475) were able to get an appointment with a physician, 5.9 % (95 % CI = 3.8–8.0 %; *n* = 35) chose not to see a physician and 14.1 % (95 % CI = 11.0–17.2 %; *n* = 84) were unable to get an appointment with a physician in the required 3-week time period. An additional 113 participants without follow-up data who received medication could be assumed to have visited a physician. Thus, at least 588 of all 893 eligible participants (65.8 %; 95 % CI = 62.7–68.9 %) visited a physician, though a somewhat higher proportion is probable due to nonresponse at follow-up by participants who had visited a physician but did not receive medication. Excluding those who chose not to make an appointment, those unable to visit a physician did not significantly differ from those who were able to visit a physician on any demographic characteristic except age, with those unable to get an appointment being younger than those able to get an appointment, 38.35 (SD = 12.11) vs. 44.14 (SD = 12.38), *p* < 0.001.

Of those eligible for study medication, 58.7 % (95 % CI = 55.5–61.9 %; *n* = 524) received either prescription medication. Almost equal proportions received varenicline (50.6 %; *n* = 265) or bupropion (49.4 %; *n* = 259). All medication packages were successfully delivered, with only 1.1 % (95 % CI = 0.2–2.1 %; *n* = 6) requiring address clarification or reshipment. When a choice of medication was offered, participants chose varenicline (85.1 %; 95 % CI = 80.0–90.1 %; *n* = 165) more often than bupropion (14.9 %; 95 % CI = 9.9–20.0 %; *n* = 29). Once the available supply of varenicline was depleted at the end of May, only bupropion was available.

Among those who received medication, 6.3 % (95 % CI = 4.2–8.4 %; *n* = 33) were initially ineligible but had altered their responses on resubmission to subsequently become eligible. The most frequent reason (more than one could apply) among these individuals for being ineligible on original submission was not planning to quit within 30 days (54.5 %; 95 % CI = 37.6–71.5 %; *n* = 18), followed by contraindication to one of the medications (27.3 %; 95 % CI = 12.1–42.5 %; *n* = 9), not currently smoking daily and/or at least 10 cigarettes per day (15.2 %; 95 % CI = 2.9–27.4 %; *n* = 5) and technical error (6.1 %; 95 % CI = 0.0–14.2 %; *n* = 2). Contraindications initially reported included an allergy to either medication, severe liver impairment, history of brain injury and current use of bupropion, varenicline, thioridazine or an MAOI.

Follow-up data were not available and therefore reasons for not receiving either medication were unknown for 59.1 % (95 % CI = 54.1–64.1 %; *n* = 218) of the 369 participants that did not receive medication. Of the 151 who did have follow-up data, the reason for not receiving medication in the majority of cases was due to not having visited a physician (80.1 %; 95 % CI = 73.8–86.5 %; *n* = 121) and in much fewer cases due to the physician not prescribing the medication (15.9 %; 95 % CI = 10.1–21.7 %; *n* = 24) or for other reasons (4.0 %; 95 % CI = 0.9–7.1 %; *n* = 6). Reasons for not visiting a physician are presented in Table 4. The most common reason reported was not being able to arrange an appointment within 3 weeks. A small number of patients did not have a family physician or were unable to access or print the prescription form. While most patients who did not visit a physician were unable to, some voluntarily chose not to visit a physician, such as those who changed their mind about quitting or were not interested in bupropion once varenicline was no longer available.

**Table 4:** Reasons reported at follow-up for not visiting a physician to discuss prescription for study medication

Reason	*n*	Percentage (95 % CI) of those that did not visit a physician (*n* = 121)	Percentage (95 % CI) of those with follow-up data (*n* = 605)
Unable to see physician			
Could not get an appointment within 3 weeks	40	33.1 (24.7–41.5)	6.6 (4.6–8.6)
Did not have a family physician	14	11.6 (5.9–17.3)	2.3 (1.1–3.5)
Did not receive confirmation email/unable to access or print study prescription	14	11.6 (5.9–17.3)	2.3 (1.1–3.5)
Too busy to make an appointment within 3 weeks	3	2.5 (0.0–5.3)	0.5 (0.0–1.1)
Other	8	6.6 (2.2–11.0)	1.3 (0.4–2.2)
Chose not to see physician			
Champix unavailable and did not want Zyban	16	13.2 (7.2–19.2)	2.6 (1.3–3.9)
Changed their mind about quitting	11	9.1 (4.0–14.2)	1.8 (0.7–2.9)
Did not want to or unable to use either medication	8	6.6 (2.2–11.0)	1.3 (0.4–2.2)
Other	1	0.8 (0.0–2.4)	0.2 (0.0–0.6)
Unknown	6	5.0 (1.1–8.9)	1.0 (0.2–1.8)

Reasons reported during follow-up interviews at 7, 11, 15 and 41 weeks. Where the same participant reported differing reasons at separate time points, the reason provided at the earliest follow-up is reported

The median number of days between study enrollment and having a prescription filled was 10 (IQR 5–16) and for having it sent out for delivery was 14 (IQR 8–21). A median of 2 days (IQR 1–5) was required after filling the prescription to reach the participant for medication counselling and to confirm shipping address.

The amount of the medication supply used was reported by 57.6 % (95 % CI = 53.4–61.9 %; *n* = 302) of participants that received medication, who reported using the following amounts: none, 2.6 % (95 % CI = 0.8–4.5 %; *n* = 8); less than 1 week, 4.0 % (95 % CI = 1.8–6.2 %; *n* = 12); 1 to less than 4 weeks, 27.2 % (95 % CI = 22.1–32.2 %; *n* = 82); 4 to less than 8 weeks, 26.5 % (95 % CI = 21.5–31.5 %; *n* = 80); 8 to less than 12 weeks, 24.5 % (95 % CI = 19.7–29.4 %; *n* = 74); all 12 weeks, 14.6 % (95 % CI = 10.6–18.5 %; *n* = 44); and don’t know/refuse, 0.7 % (95 % CI = 0.0–1.6 %; *n* = 2).

Based on an informal collection of data by the pharmacy that was reported to investigators at the end of the study, several issues arose that the pharmacist needed to resolve in order to fill a prescription, which had the potential to cause delays. Such issues included the following: need to follow-up with physicians at the request of patients when signed prescriptions were not faxed to the pharmacy, additional counselling required when physicians did not provide information to their patient regarding the medication and needing to call a physician for recommendation when a contraindication was reported by the participant. Though a precise frequency of these occurrences was not provided, the average processing time suggests that these issues did not regularly cause extended delays. The pharmacy also reported having received expired prescription forms that could not be filled and the form was revised to clearly list the expiry date. Several patients (less than 10) called the pharmacy to request the contact information of a physician that would participate in the study because their physician had declined to participate. These patients were referred to the College of Physicians and Surgeons of Ontario to find physicians accepting new patients.

Ratings of the perceived helpfulness of the weekly motivational emails were available for 44.1 % (95 % CI = 40.9–47.4 %; *n* = 394) of participants. The mean rating on a five-point scale was 2.78 (SD = 1.36). Ratings varied widely; while 42.4 % (95 % CI = 37.5–47.3 %; *n* = 167) rated the emails as not helpful at all or low on helpfulness (1 or 2), 27.7 % (95 % CI = 23.2–32.1 %; *n* = 109) selected the midpoint on the scale and 29.9 % (95 % CI = 25.4–34.5 %; *n* = 118) rated the emails helpful (4 or 5).

### Smoking cessation outcomes

The 7-day point prevalence abstinence rate among respondents at 41-week follow-up was 16.2 % (95 % CI = 9.9–22.2 %; *n* = 22) for the group that did not receive medication, 30.3 % (95 % CI = 23.3–37.3 %; *n* = 50) for the varenicline group and 24.3 % (95 % CI = 18.1–30.5 %; *n* = 45) for the bupropion group. The abstinence rates among those who received bupropion was 20.7 % (95 % CI = 5.9–35.4 %; *n* = 6) for those who had a choice of either medication and 25.0 % (95 % CI = 18.2–31.8 %; *n* = 39) for those who did not have a choice; this difference was not statistically significant (*p* = 0.65). Abstinence rates were very similar when missing data were replaced using multiple imputation; the abstinence rate at 41-week follow-up was 17.3 % (95 % CI = 14.1–20.5 %) for the group that did not receive medication, 31.0 % (95 % CI = 27.0–35.0 %) for the varenicline group, and 23.8 % (95 % CI = 20.2–27.4 %) for the bupropion group. Those who received bupropion and had a choice of medication had a quit rate of 21.5 % (95 % CI = 6.6–36.5 %) and those without a choice had a quit rate of 24.3 % (95 % CI = 17.6–31.0 %); this difference was again not statistically significant (*p* = 0.70).

### Cost analysis

The cost per patient that received medication was C$409.69. The cost per quitter ranged from approximately C$1003 (based on an overall quit rate of 24.0 % at 41 weeks among respondents only) to C$1835 (based on a quit rate of 13.1 % assuming non-respondents at follow-up were still smoking). The cost increased by approximately C$33.70 for each patient seen by a physician if they billed the government insurance plan for their services.

## Discussion

The current study establishes the feasibility of using a patient-driven model that engages smokers directly for delivery of evidence-based smoking cessation treatment including prescription pharmacotherapy and behavioural intervention. The majority of participants learned about the study through family and friends or formal communication about the study targeted to participants (e.g. posters and study website) rather than through healthcare providers, demonstrating that the decision to use and prescribe medication to quit smoking was often prompted by the patient themselves. To our knowledge, few physicians declined to participate and only a small proportion of participants who visited a physician were not prescribed the medication, suggesting a high level of acceptability of the intervention among physicians. This also suggests that screening and excluding participants based on contraindications to either medication successfully reduced unnecessary visits to the physician by those for whom the medication was not suitable. A subsample of participants reported having learned about the study from a physician, though it is unknown if this was due to study recruitment efforts directed at healthcare providers or if the impact on practice extended such that physicians who had heard about the study from patients then recommended medication to other patients. Further research is needed to determine to what extent this type of patient-mediated intervention has the capacity to change physician practice across patients and over time.

The intervention was able to reach participants from a wide range of communities across Ontario, and the only region appreciably under-represented was the city of Toronto, possibly due to a higher level of access to services. The majority of the sample that responded to follow-up was able to visit a physician to have the prescription signed within the required time period. The only demographic characteristic predictive of whether an appointment with a physician could be scheduled was age; greater difficulty getting an appointment may be due to lower rates of having a regular medical doctor among younger Canadians [38]. Very few technical difficulties were reported with accessing or printing the prescription, pharmacists were able to reach participants by telephone in a timely manner for medication counselling, and all medication packages were successfully delivered. The amount of medication used varied, though reasons for using less than the 12-week supply was not available. While some likely stopped the medication due to adverse effects or perceived lack of efficacy, some participants may have started the medication later than the study’s assumed start date for treatment and may ultimately have used the entire supply.

For the most part, safety checks were successful and the majority who reported contraindications did not go on to receive medication. Though nine individuals who initially reported medical contraindications resubmitted an assessment and did go on to receive medication, we cannot presume that the medication was prescribed in error. For example, contraindications for bupropion and varenicline were assessed jointly and thus an individual may have been prescribed the medication to which they had no contraindication. Furthermore, a physician with a more detailed knowledge of the patient’s medical history may have used their clinical judgment and deemed the medication acceptable. Individuals who initially reported using one of bupropion, varenicline, thioridazine or an MAOI may have been seeking a free supply of medication they were already using. A list of medication contraindications may be beneficial to include in the documentation provided to physicians to further ensure they screen for all possible contraindications rather than rely solely on the decision of the study regarding eligibility for medication or on the pharmacist.

More often, altering responses to fit non-medical study eligibility criteria (e.g. target quit date) occurred. These resubmissions were identified post hoc by means of a thorough examination of the personal information provided (i.e. names and birth date). Ideally, automated a priori procedures would be in place to block resubmissions and prevent prescriptions from being generated and printed. In theory, this could be accomplished by programming to detect and prohibit multiple submissions based on an identifier, such as participant name or Internet protocol (IP) address [39]. However, there are inherent limitations with such strategies. Identifying information that is manually entered is easy to alter, which we observed in many resubmissions. While a correct name is needed to fill a prescription and not as liable to falsification, we did encounter unique individuals with the same first and last names. Therefore, blocking submissions based on a combination of two separate identifiers would be beneficial, to minimize the chance of preventing legitimate submissions. Limiting submissions based on IP address or by using cookies also has limitations [39], including preventing individuals in the same household from participating [39] and trying to quit smoking simultaneously. However, in the current study, it is also possible that alteration of quit date may have demonstrated that the offer of free medication was a motivator for change, prompting an earlier quit attempt, rather than an attempt to falsify information.

Overall, findings from the current study support the feasibility of using this distribution method in a future larger trial to establish the effectiveness of this intervention for improving quit rates. A randomized trial is required in order to conclude that either medication was superior to the control condition, particularly given that the reasons for not receiving medication were largely unreported due to low response rate in that group. Given the possibility of selection bias, randomization is also necessary to make conclusions regarding the relative effectiveness of bupropion versus varenicline. While there was clearly a strong preference for varenicline in the current sample, this was likely due to the relative novelty of the medication at the time in Canada. Estimates of treatment effect obtained in the current study will be used to ensure that a larger randomized trial is sufficiently powered.

One factor to consider for future implementation is the length of time provided before the prescription is considered void. Extending the prescription expiry date may be warranted as not being able to make an appointment within the required 3-week time period was the most common reason for not having visited a physician, though overall it was not reported frequently. However, further research is needed to determine whether extending the period does in fact lead to an appreciable increase in visits to a physician. Extending the initial period may require adjustments to the methodology to take into account greater variability between participants in the length of time between enrollment and the start of treatment. One option may be to have the pharmacist log in online to indicate when a prescription has been filled to activate the start of motivational emails and determine the timing of follow-ups rather than being based on enrollment date. In addition, consideration on how to manage supply and capacity to fill prescriptions is required in future implementation of this method. While the current study managed the limited medication supply by periodically closing down enrollment due to uncertainty regarding the proportion of eligible participants that would fill a prescription, further pilot testing may be beneficial as this rate is likely to differ in other jurisdictions, using other recruitment methods etc. Placing a limit on the number of enrollments per day or week is a potential alternative strategy to shutting enrollment down completely. That adjunct motivational emails were not predominantly rated very helpful is similar to previous findings [40]. Additional research may determine how to improve the benefit to participants by altering the content or format of the messages or making these emails an optional component of the intervention.

Some limitations are noted with respect to the design of this distribution method. While online access affords an efficient, cost-effective and convenient format for assessing eligibility and enrolling in a program, its reach is limited to those who have access to the Internet and thus may be less accessible to lower-income households. In 2012, 83 % of Canadian households had access to the Internet at home; however, this proportion drops to 58 % in homes with household incomes in the lowest quartile (≤$30,000/year) [41]; however, this may be mitigated by access to the Internet outside the home such as in public libraries and community centres. Reach is also poorer in rural areas [41] and among older adults, who are less likely to be online [42].

Additional limitations of the current feasibility study existed. The response rates were very poor during the 12-week treatment period, and future studies may benefit by providing an incentive to improve response rate. The response rate did considerably increase at 41-week follow-up without any change to our methodology; the reason for this is unclear though we speculate that seasonality may partially account for this finding as surveys throughout treatment were largely conducted during the summer months. The response rate among those who did not receive medication remained relatively low though and thus the reasons for not receiving medication remains unknown for a large proportion of this group. This loss to follow-up may have led to underestimation of barriers to participation and additional barriers that went unreported may exist. Furthermore, significant differences between respondents and non-respondents indicate the possibility of bias in the reported outcomes. Findings may also not be generalizable to settings without a publicly funded healthcare system and universal access to a physician. Abstinence was not biochemically verified in the current study. A future trial designed to assess effectiveness of this intervention could verify abstinence by collecting saliva samples through the mail to determine salivary cotinine levels [43]. Finally, without knowing the number of smokers to whom information about the study was disseminated, the fact that we were able to recruit 500 eligible smokers within 6 months is not an entirely informative indicator of the success of our recruitment efforts. That is, it does not tell us what proportion who heard about the study attempted to enroll.

While an Internet-based pharmacy was shown to be an effective and efficient means to distribute prescription medication over a wide geographic area in the current study, recent legislative changes have authorized pharmacists in some jurisdictions to prescribe varenicline and bupropion for smoking cessation. This presents the option of a pharmacist distributing medication directly to the patient without the need to visit a physician. The availability of pharmacists, often without the need for a scheduled appointment, could enhance the accessibility of the intervention and dispensing in-person would eliminate delivery costs. The feasibility of this method of distribution is supported by our previous experience facilitating pharmacy-based smoking cessation interventions offering NRT and counselling [29].

## Conclusions

The current study supports the feasibility of an innovative method of distributing free prescription medications to smokers whereby those interested in quitting complete an online assessment, bring a prescription to their physician to assess the suitability of the medication, which is then dispensed and delivered by an Internet-based pharmacy. It demonstrates that using Internet-based pharmacies with the capability to dispense by mail can be used to distribute prescription medications for smoking cessation to smokers across vast geographic areas, including both major urban centres and smaller underserved rural communities to reduce geographic disparity in access to services. This method of distribution maintains some of the advantages of Internet-based treatment delivery, including increased efficiency and lower cost, but overcomes a limitation by integrating in-person clinical contact with a physician and brief telephone counselling with a pharmacist. The current model can readily be scaled up for state- or nationwide implementation, including in jurisdictions where cessation medication costs are covered by governmental health agencies, in an effort to enhance smoking cessation rates at a population-wide level.

## Additional files

Additional file 1:
**Screenshot of study website (home page).** A screenshot of the home page of the study website (stopstudy.ca) during recruitment (July 2009). (PNG 130 KB) Additional file 2:
**Letter to physician and prescription form.** Letter to the physician that provided information about the study and personalized prescription form for study medication that were personalized based on information provided in the initial assesment and sent electronically to participants to print and bring to their physician within three weeks of enrollment. (PDF 177 KB)
