# Supplementary figures and images for: Empowering smokers with a web-assisted tobacco intervention to use prescription smoking cessation medications: a feasibility trial
Source: Implement Sci. 2015 Oct 1;10:139. doi: 10.1186/s13012-015-0329-7 (PMC4590254; doi:10.1186/s13012-015-0329-7)

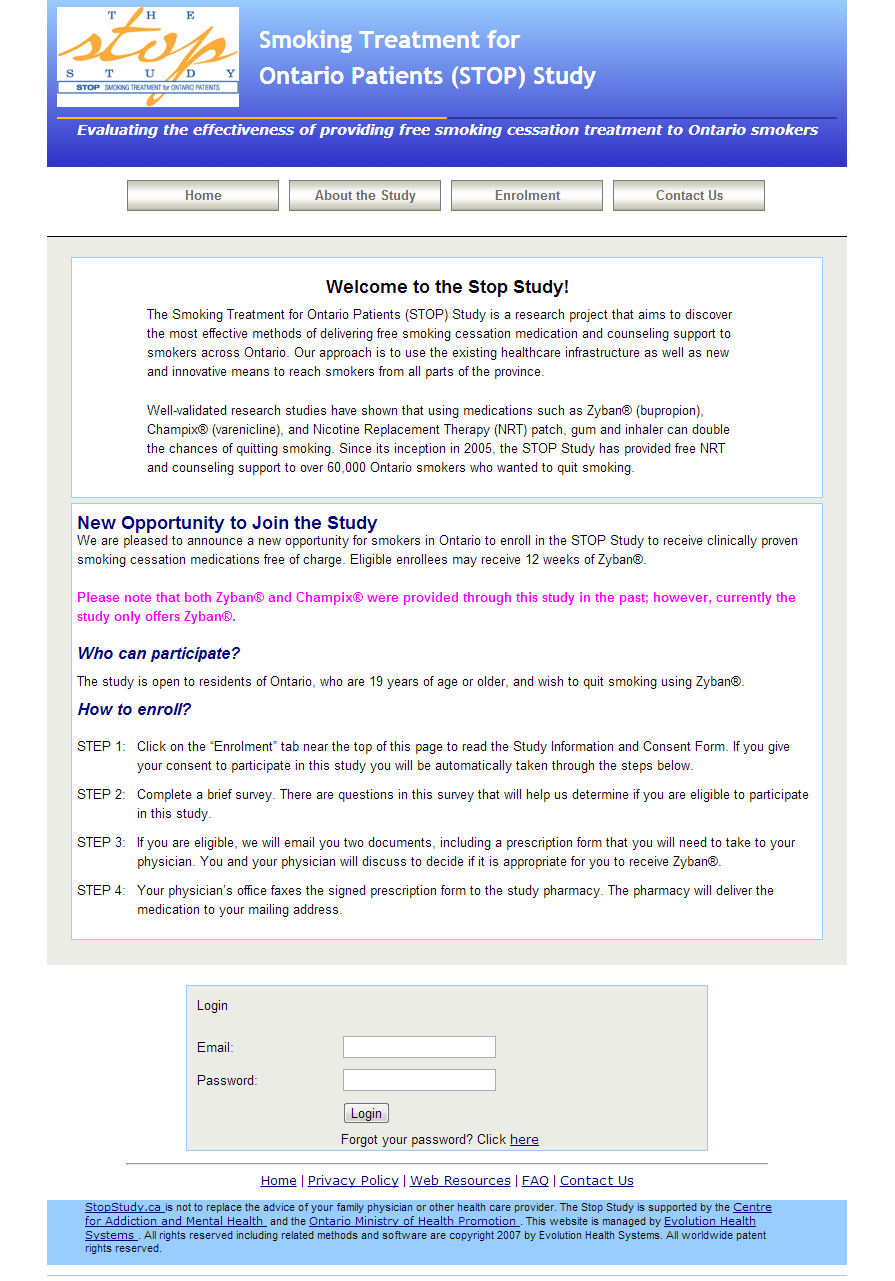

Supplement: Additional file 1: — Screenshot of study website (home page). A screenshot of the home page of the study website (stopstudy.ca) during recruitment (July 2009). (PNG 130 KB) [file 13012_2015_329_MOESM1_ESM.png]
