# Supplementary material for: Empowering smokers with a web-assisted tobacco intervention to use prescription smoking cessation medications: a feasibility trial
Source: Implement Sci. 2015 Oct 1;10:139. doi: 10.1186/s13012-015-0329-7 (PMC4590254; doi:10.1186/s13012-015-0329-7)
Supplement: Additional file 2: — Letter to physician and prescription form. Letter to the physician that provided information about the study and personalized prescription form for study medication that were personalized based on information provided in the initial assesment and sent electronically to participants to print and bring to their physician within three weeks of enrollment. (PDF 177 KB) [file 13012_2015_329_MOESM2_ESM.pdf]

Date: [*automatically populated with the date on which the participant enrolls in the study*]

RE: Your patient's decision to participate in a research study and the action requested from you

**Study Title:**	An innovative approach to maximizing the impact of efficacious pharmacotherapies on the smoking cessation attempts.
**Objectives:**	1. Explore the logistic feasibility of mass distributing prescription medication for smoking cessation across a wide geographic area (i.e., province of Ontario)<br>2. Assess the cessation rates associated with bupropion and varenicline treatment in a real-world setting, outside clinical trials
**Design:**	Open-label, self-selected intervention
**Intervention:**	12 weeks of bupropion or varenicline or neither (when doctor decides not to prescribe) plus weekly motivational emails
**Significance:**	If the method of distributing bupropion and varenicline being investigated in this study is proven to be logistically feasible and effective in terms of cessation rates, it would provide an innovative way to target and substentially reduce the overall prevalence of smoking in Ontario.
**Investigators:**	Dr. Peter Selby (Principal Investigator), Dr. Laurie Zawertailo (Co-Investigator)
**REB/IRB:**	This study has been approved by Centre for Addiction and Mental Health (CAMH) Research Ethics Board

Dear Physician,

This is to inform you that your patient, [*automatically populated with participant name*] has chosen to participate in the aforementioned research study. According to the study's eligibility criteria the patient has qualified for the study; however, the protocol resigns to your discretion to prescribe either of these medications to this patient. As the prescribing physician, the study intends to fully defer to the patient-doctor relationship and thus leave the patient under your clinical care. The study is beneficial to your patient as it offers 12 weeks of Zyban or Champix free of charge. The medication is delivered to the patient via mail from Pharmacy.ca. It is necessary for Pharmacy.ca to receive a signed prescription from you; please use the enclosed Study Registration Form.

We have embarked on a number of tobacco control initiatives ranging from research to training. If you wish to learn more about these projects or have questions or comments about this particular study please feel free to contact us.

Sincerely,

Dr. Peter Selby
Clinical Director, Nicotine Dependence Clinic
Centre for Addiction and Mental Health
peter_selby@camh.net
T: 416-535-8501 ext.6859

Dr. Laurie Zawertailo
Scientist, Clinical Neuroscience
Centre for Addiction and Mental Health
laurie_zawertailo@camh.net
T: 416-535-8501 ext.7422

STUDY REGISTRATION FORM

Study Title: An innovative approach to maximizing the impact of efficacious pharmacotherapies on smoking cessation attempts

Principal Investigator: Dr. Peter Selby

Institutional Affiliation: Centre for Addtiction and Mental Health (CAMH), 175 College St. Toronto, ON M5T 1P7 (416) 535-8501 ext. 7400

This form is void after:

[automatically populated with date 3 weeks + 1 day from date of enrollment]

PATIENT NAME [automatically populated with name provided in initial assessment]
PATIENT'S MAILING ADDRESS [automatically populated with address provided in initial assessment]<br>*Street No. Street Name: Apt: P.O. Box:*<br>*City: Province: Postal Code:*
Patient's Daytime Phone Number: [automatically populated from initial assessment]<br>*Patient's Evening Phone Number: [automatically populated from initial assessment]*

R_x

☐ Bupropion SR 150 mg for 12 weeks. Take 1 tablet once daily for first three days, then twice daily for the remainder of 12 weeks.

OR

☐ Varenicline tartrate for 12 weeks. Take 0.5 mg once daily for first three days, then 0.5 mg twice daily for next four days, then 1 mg twice daily for the remainder of 12 weeks.

MAY CAUSE DROWSINESS. ALCOHOL MAY INTENSIFY EFFECT. AVOID DRIVING VEHICLES AND OPERATING MACHINES UNTIL REASONABLY CERTAIN THAT MEDICATION DOES NOT AFFECT YOUR MENTAL ALERTNESS OR PHYSICAL COORDINATION.

Signature M.D.		*DATE*	
CPSO #	*NAME*	*ADDRESS*	*TELEPHONE #*

Must fax signed copy of this form from physician's office to the research Pharmacy:

Pharmacy.ca Fax: 1-800-727-9203
Phone: 1800-727-5048
